# Large-scale salmonella outbreak associated with banh mi, Viet Nam, 2024

**DOI:** 10.5365/wpsar.2024.15.3.1168

**Published:** 2024-09-04

**Authors:** Tinh Huu Ho, Phuong Hoai Hoang, Lam Vo Thi Ngoc, Minh Nguyen Dinh, Dong Do Thanh, Viet Nguyen Dinh, O Phan Van, Phuong Nguyen Thi Lan, Thanh Nguyen Quoc, Nhan Ho The,, Nhan Le Dinh Trong, Chinh Van Dang

**Affiliations:** a Institute of Public Health, Ministry of Health, Ho Chi Minh City, Viet Nam.; b Dong Nai Department of Health, Bien Hoa City, Dong Nai, Viet Nam.; c Dong Nai Food Safety Department, Bien Hoa City, Dong Nai, Viet Nam.; d Long Khanh Regional General Hospital, Long Khanh City, Dong Nai, Viet Nam.

## Abstract

**Objective:**

To investigate the cause of a foodborne outbreak that occurred in Dong Nai province, Viet Nam, in 2024, and implement control measures.

**Methods:**

An initial investigation was conducted to confirm the outbreak, which was followed by epidemiological and environmental investigations to find the plausible causative food item. Clinical specimens and food samples were tested to identify the pathogen.

**Results:**

A total of 547 symptomatic cases were recorded, of whom two were in severe condition requiring extracorporeal membrane oxygenation and ventilation, one of whom died. Among 99 interviewed cases, the mean incubation time was 9 hours (range 2–24 hours), with the main symptoms being fever, abdominal pain, diarrhoea and vomiting. All patients had eaten banh mi from a local bakery. *Salmonella* spp. were identified in food samples and clinical specimens. The bakery halted production, and the outbreak ended after 1 week.

**Discussion:**

All the patients were exposed to only one food in common, which facilitated the investigation process. This outbreak is a reminder to small retailers and take-away shops of the importance of food safety management in preventing similar future outbreaks. All food handlers must comply with food hygiene principles, especially in hot temperatures, which boosts bacterial growth.

Globally, *Salmonella* spp. are the most common causes of foodborne illness, leading to approximately 600 million cases and 420 000 deaths annually. (*1*) *Salmonella* spp. are the leading bacterial cause of foodborne illness in United States of America, with an annual average of 19 000 hospitalizations and 380 deaths. (*2*) *Salmonella* spp. are also the cause of 70–80% of bacterial foodborne illnesses in China, ranking as one of the country’s top two diarrhoea-causing agents. (*3*)

Salmonellae are classified into two species that can cause human illness: *S. enterica* and *S. bongori*. Salmonellae are also further subdivided into serotypes, which differ in their natural reservoirs and ability to cause human infections. (*4*, *5*) However, only a small proportion of the over 2500 serotypes cause most human infections. (*6*) Poultry and poultry products, especially eggs, are commonly linked to *Salmonella* spp. Eggs may be infected via vertical (transovarian) transmission or horizontal (trans-shell) transmission. (*7*)

Salmonellae infection most commonly occurs when a person eats contaminated food, but it can also be spread by infected persons through the faecal-oral route. The infectious period ranges from several days to several weeks. A temporary carrier state occasionally continues for months, especially in infants. Approximately 1% of infected adults and 5% of children aged < 5 years may excrete the organism for more than 1 year. (*5*, *8*)

In Viet Nam, foodborne illness affected 3711 individuals from March 2020 to August 2022. (*9*, *10*) Large-scale outbreaks related to *Salmonella* spp. have occurred in recent years. (*11*, *12*) In 2022, Khanh Hoa province reported 648 cases of school-related foodborne illness, with 211 hospitalizations and one death. (*11*) Another outbreak in Khanh Hoa was reported in 2024, with 345 cases related to contaminated chicken rice at a restaurant. (*12*) In May 2024, a suspected foodborne outbreak occurred in Dong Nai province in southern Viet Nam. The Dong Nai Food Safety Department (FSD) cooperated with the Institute of Public Health in Ho Chi Minh City (IPH) in investigating the event to determine the cause of the outbreak and implement control measures.

## Methods

The investigation was conducted from 1 to 3 May 2024, in three stages: 1) an initial investigation to confirm the outbreak; 2) an epidemiological investigation to identify foods possibly implicated in the outbreak, including investigations at food facilities; and 3) a laboratory investigation to identify the pathogen. In the epidemiological investigation, the investigation team used only descriptive statistics because all patients had eaten banh mi bought from a local bakery, and therefore matching a control group for these cases was not possible. In addition, the bakery was a take-away shop and approaching all buyers would have been a challenge.

### Initial investigation

A regional hospital in Dong Nai province admitted sporadic cases of gastrointestinal infections on the evening of 30 April 2024. As a foodborne outbreak was suspected, the hospital alerted Dong Nai FSD on 1 May. The same day, 201 other cases were hospitalized.

On the morning of 1 May, a rapid response team from Dong Nai FSD began investigating. Most patients shared similar symptoms, including diarrhoea, nausea, vomiting, abdominal pain and fever. All patients had eaten banh mi from the bakery in Long Khanh City. Based on initial information, the investigation team confirmed a foodborne outbreak and proposed to the local authority that the bakery be temporarily closed. The bakery immediately suspended its business.

### Epidemiological investigation

First, a case definition should have been established, but because laboratory results were unavailable at this phase, it was not applied. The investigation team identified banh mi as a causative agent during this outbreak. The team focused on determining the incubation period and main symptoms for potential etiology. The suspected case definition was an inpatient who ate banh mi from the bakery on 30 April or 1 May 2024, and manifested at least three of four symptoms: abdominal pain, diarrhoea, vomiting and fever.

An adapted questionnaire was designed to collect information on hospitalized patients’ characteristics, including name, sex, age, address, onset date and time, symptoms and food consumption history. The investigation team interviewed cases at the regional hospital to understand events and generate causal hypotheses. The hospital collected 23 patient faecal samples for testing at the IPH laboratory. The local authority also instructed hospitals and clinics to record all suspected patients involved in the event. Due to time and human resource shortages, the team selected 99 cases who met the case definition for interviewing. Descriptive statistics were applied to summarize information on patients, including frequency, percentage, median, mean and standard deviation.

### Environmental investigation

The investigation team assessed the bakery. It was a take-away shop that only sold banh mi. All four vendors and food handlers were interviewed about food processing, routine selling activities and their history of illness. The team observed where the food was handled. The source of all materials was also recorded.

The team took two faecal and four pharyngeal samples from the bakery staff and six food samples (pate, pork, ham, pickled vegetables and two chicken eggs).

### Laboratory investigation

Based on the symptoms and incubation time, the investigation team suspected the pathogen was a bacterium, specifically *Salmonella* spp., *Staphylococcus aureus*, Staphylococcal enterotoxins or *Bacillus cereus*. Thus, the laboratory was advised to prioritize these tests.

The IPH laboratory tested all samples using bacterial culture techniques and polymerase chain reaction (PCR) techniques. All 25 faecal samples were tested for *Salmonella* spp. (HD.PP.21–01/TT.VS). Five food samples (except one chicken egg sample) were tested for the presence of coliforms (AOAC 991.14), *Bacillus cereus* (AOAC 980.31), *Staphylococcus aureus* (AOAC 2003.07), *Salmonella* spp. (ISO 6579–1:2017), *Listeria monocytogenes* (ISO 11290–2:2017), *Clostridium perfringens* (AOAC 976.30) and Staphylococcal enterotoxins (ISO 19020:2017). The remaining chicken egg sample was only tested for *Salmonella* spp. The four pharyngeal samples were tested for the presence of coliforms (HD.PP.21–01/TT.VS), *Salmonella* spp. (HD.PP.21–01/TT.VS, HD.PP.10–05/TT.VS) and *S. aureus* (HD.PP.21–01/TT.VS). Results under 10 cfu/g were considered undetectable.

## Results

### Epidemiological investigation

A total of 547 cases of foodborne illness were recorded from 30 April to 6 May 2024. Of those, 52% were female. The average age was 35.0 years, and the age distribution was 29% aged < 20 years, 46% aged 20–49 years and 25% aged ≥ 50 years (**Table 1**). All patients resided in Long Khanh City. The regional hospital received 497 patients (90.9%, while six hospitals received the others (**Table 1**).

**Table 1:** Characteristics of recorded patients in foodborne outbreak in Dong Nai province, Viet Nam (*n* = 547)

Characteristic	n	%
**Sex**		
**Male**	**263**	**48.1**
**Female**	**284**	**51.9**
**Median age (years)**	**35**	**-**
**Mean age (years) (mean ± SD)**	**34.4 ± 19**	**-**
**Age group (years)**		
** < 20**	**159**	**29.1**
**20–49**	**252**	**46.1**
** ≥ 50**	**136**	**24.9**
**Hospital**		
**Regional**	**497**	**90.9**
**Other**	**50**	**9.1**

**Fig. 1:**
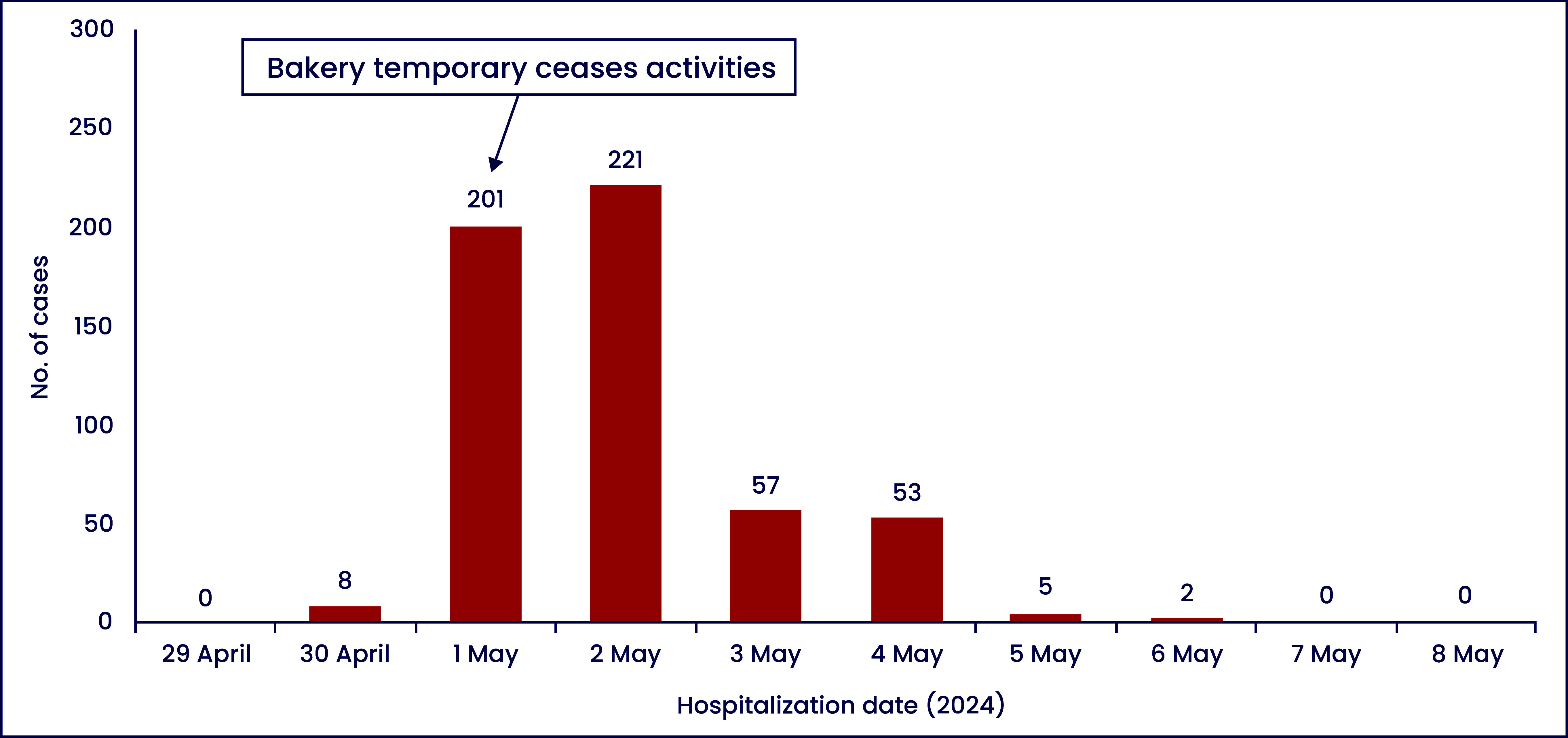
Epidemic curve of 547 foodborne illness cases in Dong Nai province, Viet Nam, 2024

After temporarily ceasing the activities of the bakery on the morning of 1 May, the number of recorded cases peaked on 2 May and then sharply decreased in the following days, ending on 7 May (**Fig. 1**). Based on the epidemic curve, this was a point-source outbreak, lasting from 30 April to 7 May.

[**insert** Figure 1]

Among the 99 interviewed patients, the average age was 36.2 years, and cases appeared in all age groups. The most prevalent symptoms were diarrhoea (90/99, 90.9%), abdominal pain (80/99, 80.8%), fever (65/99, 65.7%) and vomiting (58/99, 58.6%). The average incubation time was 9 hours (**Table 2**). Two patients in severe condition required extracorporeal membrane oxygenation and ventilation. After a few weeks of treatment, one case recovered and the other died.

**Table 2:** Characteristics of interviewed patients in foodborne outbreak in Dong Nai province, Viet Nam (*n* = 99)

Characteristic	n	%
**Sex**		
**Male**	**41**	**41.4**
**Female**	**58**	**58.6**
**Median age (years)**	**35**	**-**
**Mean age (years) (mean ± SD)**	**36.2 ± 20.0**	**-**
**Age group (years)**		
** < 20**	**27**	**27.3**
**20–49**	**43**	**43.4**
** ≥ 50**	**29**	**29.3**
**Symptoms**		
**Diarrhoea**	**90**	**90.9**
**Abdominal pain**	**80**	**80.8**
**Fever**	**65**	**65.7**
**Vomiting**	**58**	**58.6**
**Nausea**	**6**	**6.1**
**Incubation time (hours)**		
**Mean ± SD**	**10.1 ± 5**	**-**
**Median (range)**	**9.0 (2.0–24.0)**	**-**
**Severe condition**	**2**	**2.0**
**Death**	**1**	**1.0**

SD: standard deviation.

### Environmental investigation

The bakery had sold banh mi for more than 20 years. Products were sold daily at 6:00–9:00 and 15:00–19:00, averaging about 1000 banh mi sales daily. The staff estimated that around 1500 banh mi were sold from 30 April to the morning of 1 May. Banh mi is a Vietnamese baguette sandwich filled with pate, Vietnamese pork roll, ham, pork and pickled vegetables. The bakery made the pate, pickled vegetables and sauces. The remaining foods were bought from a third-party supplier.

The investigation team observed that the bakery did not follow the one-way principle for food processing. All the processes overlapped in the cooking stages, and collisions or contact between raw and cooked foods could occur. The areas for preparing raw and cooked foods were next to each other, and there was no table or food storage shelf. The food and raw materials were put on the floor or in two cold stores. The team could not assess the temperature of the cold storage because they had been sealed by the police. All food processing utensils (knives, cutting boards, baskets and pots) were placed together, suggesting that they could be shared in the preparation of raw and cooked foods. However, the facility owner asserted that they cleaned and used all the instruments separately. Protective gloves were not used.

### Laboratory results

*Salmonella* spp. were found in 12/25 faecal specimens (48.0%) and 4/6 food samples (66.7%). Noticeably, both faecal specimens from the staff were positive for *Salmonella* spp. Food samples were also contaminated with coliforms (3/6, 50.0%), *S. aureus* (2/6, 33.3%) and *B. cereus* (1/6, 16.7%) (**Table 3**). Salmonellae in this outbreak were of serogroup OMA among both patients and staff.

**Table 3:** Laboratory results of clinical and food samples

Type of sample	Positive results			
	*Salmonella* spp.	*Staphylococcus aureus*	Coliforms	*Bacillus cereus*
**Faecal samples**				
**Patients (***n* **= 23)**	**10 (43.5)**	**N/A**	**N/A**	**N/A**
**Staff (***n* **= 2)**	**2 (100.0)**	**N/A**	**N/A**	**N/A**
**Pharyngeal samples**				
**Staff (***n* **= 4)**	**0**	**2 (50.0)**	**2 (50.0)**	**N/A**
***Food samples (n = 6)^a^***	**4 (66.7)**	**2 (33.3)**	**3 (50.0)**	**1 (16.7)**

## Discussion

Banh mi was the source of this community-based foodborne outbreak in Dong Nai province. The incubation time suggested a bacterial pathogen. *Salmonella* spp. were detected in both food and faecal samples. Fever, abdominal pain and diarrhoea are the most prevalent symptoms of salmonellosis, and the incubation period is 6–72 hours (usually 12–36 hours). (*5*, *13*) Thus, salmonellae was the most plausible agent of this outbreak.

This salmonellosis outbreak had a considerable number of cases (547 hospitalizations), out of which two were severe and one died. The causative food was sold by a take-away bakery that, between 30 April and the morning of 1 May 2024, had sold an estimated 1500 portions, meaning up to 1500 people could have been infected. The 547 hospitalized cases accounted for 36.5% of this at-risk group. The hospitalization rate was in line with the 2022 European Centre for Disease Prevention and Control (ECDC) surveillance report of 39.3% out of 29 712 patients hospitalized. (*14*) The case fatality rate in this outbreak was 0.18% (1/547), which did not greatly differ from the ECDC report of 0.22%. However, the fatality rate in the United States was estimated to be higher at 1.6% (420 deaths among 26 500 hospitalizations annually). (*15*)

Most food samples (66.7%) were positive for *Salmonella* spp., indicating that all foods could be cross-contaminated due to poor hygiene practices. The results of the environmental investigation supported this assumption. The lack of a one-way approach to food processing and using the same utensils to process raw and cooked foods could be crucial reasons for cross-contamination. (*16*) The absence of a table or shelf for putting food and not wearing protective gloves could be additional reasons.

The time of year during the outbreak is the hottest in Dong Nai province, with daily average temperatures around 37–38 °C. This is optimal for the growth of *Salmonella* spp. (*17*) Thus, inappropriate cooking and preserving practices carry a particularly high risk of foodborne outbreaks. Food standards do not allow for the presence of *Salmonella* spp., as a single organism could be enough to cause a foodborne event. (*18*)

Faecal specimens from the bakery staff were positive for *Salmonella* spp., and the serogroup (*Salmonella* OMA) was consistent with those of the patients. An asymptomatic carrier was possibly the cause of the outbreak. However, they could have been infected through the same sources as the patients. Thus, active surveillance of bakery staff is necessary for confirmation. Not all of the patients’ faecal specimens were positive for *Salmonella* spp. because excretion of the organisms may be intermittent, (*19*) or patients could have taken medicines before hospital admission.

Symptoms of salmonellosis usually last for a few days, and most infections are self-limiting. Severe illness can occur, especially among children, older adults and persons with chronic diseases. In this outbreak, one severe case was a 7-year-old boy who was overweight (47 kg), with congenital deafness and muteness. He had symptoms on 30 April and was admitted on 1 May with diarrhoea and abdominal pain. He was in a serious condition the next day and needed a ventilator. After a few weeks of treatment, he recovered and was discharged.

The case who died was a 6-year-old boy who was hospitalized relatively late. Symptoms manifested on 30 April, and he was treated at home with over-the-counter medicines. He became comatose and experienced convulsions and cyanosis and was in cardiac arrest when admitted on 2 May. The boy died after 1 month of intensive treatment. Therefore, delayed presentation to hospital and inappropriate first aid, especially lack of rehydration for patients with diarrhoea, (*20*) can lead to more severe outcomes.

Although the outbreak was investigated and controlled promptly, the investigation team acknowledged certain limitations. Interviewing all patients was challenging due to the limited number of team members, and many patients with mild symptoms left the hospital before the investigation commenced. The team encountered difficulties collecting information from all individuals who consumed the contaminated banh mi because the bakery operated as a take-away shop. Consequently, a cohort study and attack rate calculation could not be performed.

In this outbreak, hospitals in Dong Nai responded well based on their available emergency response plans. They were adequately supplied with the tools and drugs to treat diarrhoeal infection, especially temporary toilets for use in overcrowded conditions to prevent cross-contamination.

Viet Nam lacks annual salmonellosis data due to the absence of a specific reporting system. Implementing such a system is crucial for rapid response but demands substantial resources, particularly financial. A sentinel surveillance system could serve as an effective alternative. This outbreak highlights the risk of foodborne illness from take-away facilities and small retailers. Local authorities should manage all food facilities well and raise awareness of proper food handling practices among vendors.
